# Education and Social Support as Key Factors in Osteoarthritis Management Programs: A Scoping Review

**DOI:** 10.1155/2018/2496190

**Published:** 2018-05-08

**Authors:** Shabana Amanda Ali, Kristina M. Kokorelias, Joy C. MacDermid, Marita Kloseck

**Affiliations:** ^1^Faculty of Health Sciences, University of Western Ontario, 1151 Richmond St., London, ON, Canada N6A 5B9; ^2^Hand and Upper Limb Centre Clinical Research Laboratory, St. Joseph's Health Centre, 268 Grosvenor St., London, ON, Canada N6A 3A8

## Abstract

Systematic reviews of self-management programs for osteoarthritis suggest minimal evidence of benefit and indicate substantial heterogeneity in interventions. The purpose of this scoping review was to describe the nature of self-management interventions provided to patients with osteoarthritis focusing on the inclusion and type of education and social support components. We searched PsycINFO, EMBASE, MEDLINE, and Cochrane Library databases from 1990 to 2016 to identify studies addressing community-based management strategies for osteoarthritis that included aspects of disease-specific education and ongoing social support. Results are presented as a narrative synthesis to facilitate integration of diverse evidence. Data were extracted from 23 studies that met our inclusion and exclusion criteria, describing complex, multicomponent interventions for osteoarthritis. All studies included education components, and 18 of these were osteoarthritis-specific. Social support was most often offered through peers and health care professionals, but also through exercise trainers/instructors and researchers, and lasted between 5 and 52 weeks. We charted positive social interaction offered by peers in group settings and emotional/informational support offered by health care professionals. Overall, descriptions of self-management provided limited documentation of the rationale or content of the programs. This suggests that more precise definitions of the theoretical underpinnings, components, and mechanisms would be useful for greater insight into best practices for osteoarthritis self-management programs.

## 1. Introduction

Osteoarthritis (OA) is a chronic disease that causes pain and disability, affecting 1 in every 2 older adults over the age of 65 [1]. Self-management programs have been reported for several chronic conditions, including osteoarthritis [2, 3]. These programs are typically behavioural interventions that encourage people to take an active role in managing their own condition by providing education and management strategies [4]. A systematic review of self-management education programs for osteoarthritis found low to moderate quality evidence to suggest that these programs result in no or small benefit, but are unlikely to cause harm [5]. Importantly, only 12% of interventions addressed social integration and support. When compared to usual care, self-management education programs slightly improved osteoarthritis pain, function, and symptoms, but these benefits were not necessarily clinically meaningful [5]. Self-management programs may be particularly challenging for older adults who are managing multiple morbidities [3].

Like other chronic diseases, osteoarthritis must be managed daily, often at home within the community setting, over long periods of time [1]. Osteoarthritis can be seen as less of a priority than other chronic disease by both health care providers and patients as it is considered a non-life-threatening condition [6]. Emerging evidence indicates that osteoarthritis is associated with an increased risk of death [7, 8], but this information has yet to displace commonly held myths about osteoarthritis being an inevitable part of aging [9]. There are several strategies that can be used to mitigate symptoms of osteoarthritis, including exercise, strength training, and weight management, among others [10]. The challenge lies in effective implementation of these strategies among community-dwelling older adults [11]. Existing self-management programs that require older adults to take responsibility for managing their osteoarthritis may be lacking critical factors that facilitate adoption of disease management strategies. For example, behaviour change theory and techniques can be effective but are not always applied or reported in studies of group-based self-management programs [4].

Disease-specific interventions have been shown to be more effective than generalized chronic disease interventions, particularly for community-dwelling older adults [12, 13]. The majority of existing self-management programs for arthritis do not distinguish among the subtypes but provide general strategies for disease management of both rheumatoid arthritis and osteoarthritis [14]. Given that osteoarthritis and other arthritis subtypes have different etiologies and treatments, tailoring management programs to include disease-specific education might improve outcomes for a significant portion of the arthritis population.

Chronic disease self-management programs are more effective when social support is offered [15], and this is well documented in the diabetes literature [16, 17]. According to previous findings, when support is offered through a social network in an ongoing capacity, self-management behaviour improves. Multitrait scaling analyses using results from patients with chronic conditions in the Medical Outcomes Study [18] suggest that there are four functional social support scales which include emotional/informational support, tangible support, affectionate support, and positive social interaction. The mechanisms by which social support works may include increased self-efficacy, motivation, coping, or overall psychological wellbeing [15]. This is consistent with social cognitive theory upon which self-management programs are sometimes based, where behavioural factors (e.g., self-efficacy), environmental factors (e.g., social network), and cognitive factors (e.g., knowledge) interact to determine behaviour [4, 19].

Although a systematic review might synthesize evidence on chronic disease management, these complex interventions can be quite variable. Based on existing evidence for the benefits of disease-specific interventions and social support in chronic disease management, we conducted a scoping review to describe the state of the literature concerning these two factors in osteoarthritis management. Since a previous systematic review found that only 12% of studies addressed social integration and support [5], our objective was to provide a descriptive summary of how social support is delivered in osteoarthritis interventions, focusing on functional dimensions [20]. We also explore the disease specificity of educational components of osteoarthritis interventions. The information gathered can be used to establish areas of existing evidence and gaps that should be addressed in future studies to ultimately improve osteoarthritis management programs.

## 2. Methods

### 2.1. Identifying the Research Question

We used scoping review methodology guided by Arksey and O'Malley [21] to answer the following question:* What is known from the existing literature on community-based strategies for the management of osteoarthritis that include disease-specific education and social support?* We sought studies describing complex interventions for osteoarthritis that included* education* and* support* delivered in* community* settings.* Education*: we were interested in disease-specific education, where management recommendations were delivered to patients with osteoarthritis.* Social support*: we conceptualized social support based on two of the four functional social support scales identified in the Medical Outcomes Study [18]. We considered emotional/informational support to be offered by health care professionals, given that they provide information and advice to patients who turn to them. We considered positive social interaction to be offered by peers in group settings, given that they come together for a common activity.* Community*: we defined community as the local physical environment in which interventions could be implemented and social interactions could be sustained (e.g., personal residences, retirement communities, and community centres).

### 2.2. Identifying Relevant Studies

A literature search was conducted by two authors and was deliberately broad to gather suitable articles from PsycINFO, EMBASE, MEDLINE, and Cochrane Library databases from 1990 to 2016 using the keywords* osteoarthritis, management, education, social, support, community, *and* health*. Truncations were used to search for variant forms of the keywords. Connector words such as “and” and “or” were used to yield more search results. Hand searching of the reference lists of relevant articles was also conducted.

### 2.3. Study Selection

**Figure 1:**
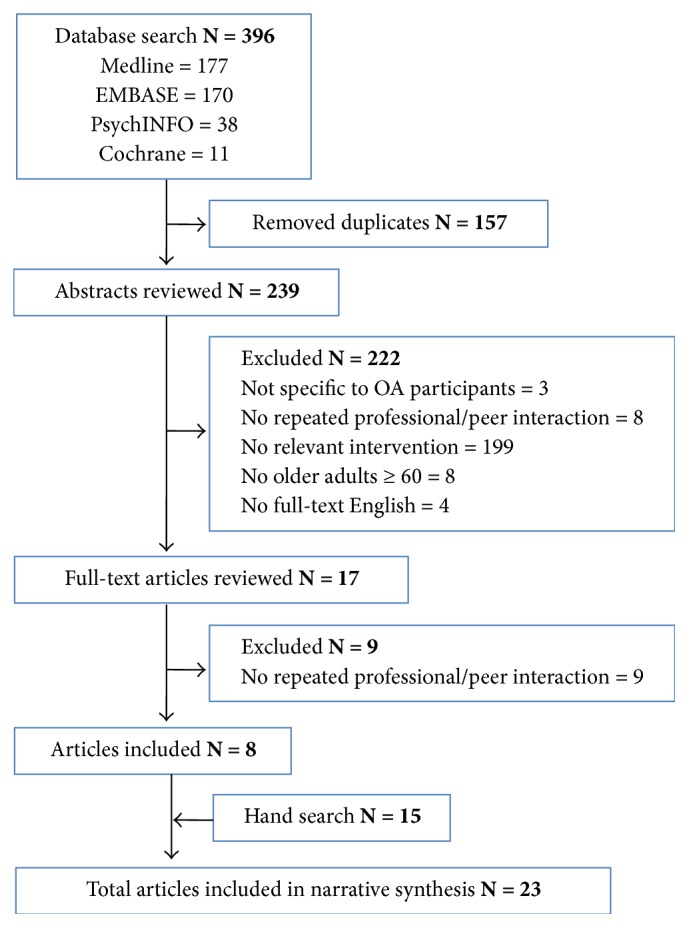
Flowchart of literature search strategy.

Studies were included if they (1) described* education* delivered to participants with osteoarthritis, (2) included repeated interactions with professionals or peer groups* (social support)*, (3) described an intervention in a* community*-based setting, (4) included older adults ≥ 60 years of age, or health care practitioners who care for older adults ≥ 60 years of age, and (5) were published in full-text, in the English language, between January 1990 and December 2016. Studies that failed to meet any one of these criteria were excluded (Figure 1). First, all articles were screened by title and abstract by two authors. Next, the remaining articles were screened by full-text to determine eligibility based on the inclusion and exclusion criteria. During this step, disagreements regarding inclusion status were resolved by discussion between two authors until consensus was reached.

### 2.4. Charting the Data

A total of 23 articles were included in this review (Figure 1). Data were extracted from each included study and organized into table format [21]. Relevant details from articles included information on study design, sample size, objectives, intervention, results, and interpretation of the findings (Supplementary Table 1). An additional table was created to summarize the design of each intervention pertaining to the community setting (location where the intervention was delivered), education (disease focus and topics covered), and type of support that was offered (social agent and duration; Supplementary Table 2).

### 2.5. Collating, Summarizing, and Reporting the Results

Consistent with scoping review methodology, we describe the extent and nature of the available evidence, but not the quality [22]. We present the results as a narrative synthesis to provide greater detail and integrate diverse evidence for an interdisciplinary target audience of researchers, practitioners, and policy-makers [21]. Results are summarized according to two major commonalities across the studies, including group-based interventions and repeated interactions with health care professionals.

## 3. Results

Our search identified 23 studies describing management strategies for osteoarthritis that offer education and support in community settings. Synthesis of our results revealed that all interventions included patients with osteoarthritis, but only 18 interventions offered osteoarthritis-specific education. Of these, 6 interventions offered joint-specific osteoarthritis education covering hip OA (*N* = 3), knee OA (*N* = 2), or hand OA (*N* = 1). The majority of education programs combined self-management strategies with exercise training (*N* = 12), while others focused on self-management alone (*N* = 5), exercise alone (*N* = 4), or pain-coping skills training (*N* = 2). Further details regarding the focus (joint-specific OA versus multiple-joint OA versus general arthritis) and topic (self-management, exercising training, or both) of education that was provided in each intervention are described in Supplementary Tables 1 and 2.

The way in which social support was incorporated into interventions is less clear. Here we categorize social support based on positive social interaction occurring among peers in group-based interventions, and emotional/informational support received from health care professionals during delivery of the interventions. These categories are not mutually exclusive, as health care professionals may be involved in delivering group-based interventions, but this was the most meaningful way to interpret social support given the lack of clear reporting on this construct.

### 3.1. Positive Social Interaction in Group-Based Interventions

Group-based interventions were described for 16 of the 23 studies included (Supplementary Table 2). A qualitative study exploring health care professionals' views on a group-based exercise suggests they consider group-based interventions to be an acceptable and feasible approach for the management of osteoarthritis [23]. Group-based interventions provide participants the opportunity to share experiences and offer support to their peers in achieving common goals, which is consistent with positive social interaction [18]. Hurley et al. design their group-based intervention to promote an increase in self-management behaviour using self-determination theory which highlights the role of “social agents in facilitating autonomous motivation and perceived competence for long-term behaviour change” [24]. Coleman et al. show that group-based delivery of a self-management program for people with knee osteoarthritis improved pain, mental health, and physical functioning that persisted for up to 12 months [25]. Several studies show that group education combined with exercise result in positive effects on self-efficacy, ultimately reducing pain and improving functional outcomes [26–28]. The Good Life with osteoArthritis in Denmark (GLA:D) self-management and neuromuscular exercise training program showed positive outcomes after 3 months and 12 months, but the differences were not explored between group education versus traditional education, nor group exercise versus home-based exercise [26, 27].

Group-based physical activity interventions allow repeated exposure to the same social network while engaging in common behaviour, and this positive social interaction can have positive effects on outcomes. Stener-Victorin et al. explored electroacupuncture, hydrotherapy, and patient education for osteoarthritis and reported that participants received the most support in hydrotherapy (group-based), followed by electroacupuncture (individual-based) and then education [29]. Both hydrotherapy and electroacupuncture, but not education alone, led to improvements in pain, aches, disability, and quality of life [29]. Kim et al. found a group-based “aquarobic” program delivered in 36 sessions over 12 weeks enhanced self-efficacy, reduced body weight, pain, and depression levels, and improved blood lipid concentrations in patients with osteoarthritis [28]. Hartman et al. offered T'ai Chi training through two 1-hour classes per week for 12 weeks and found improvements in self-efficacy, quality of life, and functional mobility among older adults with lower extremity osteoarthritis [30]. Hughes et al. offered physical therapist-led sessions three times per week for 8 weeks with 15 participants at a time and found improvements in exercise efficacy, exercise adherence, function (walking distance), and symptoms (pain, stiffness) [31]. These studies suggest that physical activity interventions are amenable to group-based delivery and can improve outcomes [32].

Group-based interventions may improve outcomes by increasing compliance compared to individual-based interventions [33]. Eitzen et al. found no effect of a 12-week supervised education and exercise therapy program on gait in patients with osteoarthritis, but the exercise component was delivered individually and resulted in low compliance while the education component was group-based and showed 100% compliance [34]. Even over the course of a year, a community-based water exercise program achieved 70% compliance for the twice-a-week group sessions [35]. Keefe et al. showed that when spouses of those with osteoarthritis were trained to reinforce their spouses pain-coping skills (through 10 weekly 2-hour sessions in groups of 4–6), participants experienced significantly reduced pain, psychological disability, and pain behaviour, plus higher scores on measures of coping attempts, marital adjustment, and self-efficacy [36]. These studies suggest that peers may reinforce behaviours for osteoarthritis patients and improve their compliance with interventions.

An additional method for positive social interaction between peers is telephone follow-up. Crotty et al. evaluated a program including a 6-week self-management course, group education, and individualized peer support phone calls, where peers were volunteer peer support educators from a local arthritis support and advocacy group [37]. They found improvements to health-directed behaviours, skill and technique acquisition, and joint stiffness, but no effect on pain, disability, quality of life, or depressive symptoms after 6 months. The authors did not discuss the impact of the peer support phone calls on outcomes despite the fact that 25 of 75 participants in the intervention group requested no further phone calls after the first one. The remaining 50 participants received an average of 5 phone calls in total, approximately one per month [37].

Determining the conditions in which positive social interaction is best delivered may have economic implications. Cronan et al. offered education, support, and both in combination over 10 weekly 2-hour meetings and 10 monthly 2-hour meetings [38]. Positive social interaction included peer group discussions to promote empathy and sharing of coping techniques between group members. Health care costs for those who received education supplemented with support were lower by $1,279 per participant per year over 3 years, compared to the control group. The support intervention had the lowest implementation cost, and when combined with education, reduced attrition [38].

### 3.2. Emotional/Informational Support from Health Care Professionals

Health care professionals were involved in the interventions for 15 of the 23 studies included (Supplementary Table 2). Given that health care professionals provide information and advice to patients who turn to them, we considered this a form of emotional/informational support [18]. Many studies were designed such that delivery of the intervention over multiple time points by health care professionals may have offered social support to otherwise isolated older adults. For example, Bennell et al. describe ten individual visits with a physiotherapist for supervised training and practice [39]. Hay et al. describe 3–6 sessions with a community pharmacist plus 3–6 sessions with a community physiotherapist over a 10 week period. The result was improvements in health outcomes, reduced use of medication, and high patient satisfaction [40]. Importantly, these improvements were noted at 3 months, but were not sustained after 6 months or 12 months when support was no longer offered.

Telephone coaching by health care professionals has been predicted to improve adherence to interventions. Brosseau et al. tested a behavioural intervention that included support through face-to-face and telephone counseling and showed lower dropout rates and higher retention rates after 12 and 18 months compared to groups without support [41]. Three other protocols describe interventions that include telephone follow-up by professionals. Bennell et al. reported a protocol with an intervention where nurse-delivered telephone coaching was offered between 6 and 12 times in 6 months [42]. Østerås et al. reported a protocol with group-based and home-based sessions, where participants received weekly telephone calls from an occupational therapist during the 8 weeks with no group sessions [43]. Moe et al. describe an intervention including a group-based education program plus individual consultations with members of a multidisciplinary team (rheumatologists, nurses, health secretaries, occupational therapists, physical therapists, pharmacists, orthopaedic surgeons, and a dietician) and follow-up at 4 months with a 10-minute phone call from a researcher [44]. Though the results of these studies were not identified through our search, their aim was to promote positive outcomes through emotional/informational support from individuals with whom patients may not otherwise interact.

When support is offered by health care professionals, one outcome may be increased use of medication. Rosemann et al. conducted a study where general practitioners delivered arthritis information to participants and a subset of participants received additional case management support through monthly phone calls from a nurse over 6 months [45]. The phone calls covered a structured questionnaire that asked about pain, effects, and side-effects of prescribed drugs, and adherence to the general practitioners' recommendations regarding physical activity. Depending on the answers, patients would be grouped by urgency into one of the following categories: immediate doctor referral, information forwarded to the doctor after the telephone call, or information forwarded at the end of the day. Regardless of the category, patients increased their salience with health care professionals through the additional case management support and this resulted in increased prescriptions of pain relievers, among other outcomes [45].

## 4. Discussion and Conclusion

### 4.1. Discussion

Charting the literature on community-based strategies for the management of osteoarthritis showed substantial diversity in the content of education and social support offered to patients. The specific design and theoretical rationale of these programs are often unclear. Scoping reviews are typically done prior to a systematic review to establish whether the literature is amenable to this type of literature synthesis. This scoping review highlights that the diversity in the literature might be problematic in systematic reviews since there is a high likelihood that interventions of different effectiveness would be combined, but the lack of description might make it difficult to differentiate sources of heterogeneity. Previous and future systematic reviews would have difficulty determining what types of interventions were most effective given the diversity in the content of the existing studies. This limits the ability to define best practice for education and support in osteoarthritis management.

Given that osteoarthritis consumes a considerable portion of health care budgets [46], there is a strong impetus to improve management in community settings and reduce costs. Patients themselves cite disease-specific education and targeted community-based programs for the management of osteoarthritis as key goals of therapy [47–49]. The low risk to participants and high potential to reduce health care costs support the use of community-based education and physical activity regimens in care plans [50]. Despite the extensive literature focused on self-management programs for arthritis dating back to 1985 [51], there is minimal beneficial effect of these programs for osteoarthritis [5]. To date, self-management programs that are education-based have shown variable results with questionable impact on clinical outcomes [5]. Even when delivered through primary care physicians, self-management education did not improve most outcomes or reduce use of health care resources [52].

We conducted this scoping review to describe the evidence on two key factors that may impact complex community-based management programs for osteoarthritis: education and social support. Our major finding was the considerable variability in the content and reporting of these interventions. Duration of social support ranged from 5 weeks to 52 weeks, providing little insight into the ideal length of time required for support. The social agent delivering support most often included peers or health care professionals or both, though there were no direct comparisons on the impact to outcomes. The format in which social support was offered was mostly in-person interactions (group-based interventions, repeated visits with health care professionals), with some studies offering telephone follow-up that is viewed as a source of social support to elderly persons who may have support deficits [53].

Given that several studies show that positive social interaction and emotional/informational support can reinforce behaviour and improve compliance, further research into social support from peers and professionals is merited. In particular, social influence and support from peers in group settings can improve compliance and adherence and offer psychosocial and sociobehavioural benefits in health care [29, 54]. The majority of group-based interventions we identified were led by health care professionals but offered opportunities for participants to interact with peers and receive reciprocal support towards achieving a common goal. It remains unclear whether the participants in the included studies truly experienced social support, in what form, and to what degree. The perceived or actual level of support that is received is unknown, as it was not directly measured in any of the studies [55]. This could be a previously unidentified factor that explains the variation in findings across studies that appear to offer similar management interventions for osteoarthritis. Future studies might include direct measures of social support for individuals with chronic conditions, such as the Medical Outcomes Study Social Support Survey Instrument [18].

Looking beyond osteoarthritis, reports describing community-based programs for the management of rheumatoid arthritis also show changes to outcomes that are variable, relatively small, and not necessarily clinically meaningful [56–58]. Morrin et al. describe a community-based management program for chronic disease that includes patient education, supervised exercise, and self-management support (through ongoing workshops, tools, and messaging with lay leaders and program staff) [59]. While this program includes social support, the education component is general to arthritis rather than specific to osteoarthritis. Our results suggest that disease-specific, and even joint-specific, education on osteoarthritis could provide information that is directly relevant to debunking myths and improving outcomes. The Good Life with osteoArthritis in Denmark (GLA:D) initiative addresses this, offering osteoarthritis-specific education focused on diagnosis, aetiology, risk factors, symptoms, treatments, and self-help [26]. Elements of social support could be maximized in this program through increased group-based education and exercise [27].

### 4.2. Limitations

We were unable to conclude on clear parameters of osteoarthritis-specific education and social support (e.g., format, duration, and social agent) needed to produce clinically meaningful changes in outcomes. This limitation may be common to understanding complex interventions where it is difficult to isolate the active ingredient contributing to outcome effects. We presented results as a narrative synthesis in order to describe the diversity in evidence, but we were unable to evaluate superiority of the various intervention components. We were also unable to determine the perceived or actual support received by participants in various interventions [55] and therefore were unable to determine potential mechanisms underlying the effect of ongoing support on disease outcomes [60]. We conceptualized social support using the Medical Outcomes Study categories but recognize that there are other taxonomies for operationalizing this construct [55]. We recognize advancements in Internet-based interventions [61–63] but did not include them in this review. We focus on in-person interactions given that this is the predominant delivery method for current osteoarthritis management programs.

### 4.3. Conclusion

This scoping review on community-based strategies for the management of osteoarthritis showed substantial diversity in the education and social support offered. Education delivered to osteoarthritis patients ranged in disease specificity (e.g., specific to osteoarthritis versus general to arthritis) and joint specificity (e.g., hip versus knee versus lower extremity). Support varied in terms of format (in-person versus telephone follow-up), duration (5 to 52 weeks), and the social agent (peers versus health care professionals). Variation in interventions across studies, including a lack of detailed description of the theoretical basis and limited information on content and delivery of the program components, makes it challenging to determine the relative contribution of each component in these complex interventions. There is a need for future studies to test more clearly defined disease-specific education with integrated social support in community settings, so that critical components can be established and future systematic reviews will be able to test for clinical heterogeneity.

## Supplementary Materials

Supplementary 1 Supplementary Table 1: summary of included studies by study design, sample size, objectives, intervention, results, and interpretation.

Supplementary 2 Supplementary Table 2: summary of included studies by community setting, education focus, and social support.
