# Supplementary material for: Education and Social Support as Key Factors in Osteoarthritis Management Programs: A Scoping Review
Source: Arthritis. 2018 May 8;2018:2496190. doi: 10.1155/2018/2496190 (PMC5964569; doi:10.1155/2018/2496190)
Supplement: Supplementary 1 — Supplementary Table 1: summary of included studies by study design, sample size, objectives, intervention, results, and interpretation. [file 2496190.f1.docx]

**Supplementary Table 1.**Summary of included studies by study design, sample size, objectives, intervention, results, and interpretation.

| Authors | Study Design | Sample Size | Objectives | Intervention | Results and Interpretation |
| --- | --- | --- | --- | --- | --- |
| Alkatan et al. (2016)  PMID  26541906 | Randomized controlled trial | N=48 | To determine whether regular swimming reduces vascular dysfunction and inflammation and elicits similar benefits compared with land-based exercises such as cycling in terms of reducing vascular dysfunction and inflammation in patients with OA. | Randomly assigned to swimming or cycling training groups. Cycling training was included as a non-weight-bearing land-based comparison group. 12 weeks of supervised exercise training. | Regular swimming exercise can exert similar or even superior effects on vascular function and inflammatory markers compared with land-based cycling exercise in patients with OA. |
| Bennell et al. (2012)  PMID  22828288 | Randomized controlled trial protocol | N=70 per arm | To investigate whether an integrated 12-week PCST and exercise treatment program delivered by physiotherapists is more efficacious than either program alone in treating pain and physical function in individuals with knee OA. | 12-week intervention involving 10 physiotherapy visits together with home practice - randomized into one of three groups: exercise alone, PCST alone, or integrated PCST and exercise. | Primary outcomes are overall average pain in the past week. Secondary outcomes include global rating of change, muscle strength, functional performance, physical activity levels, health related quality of life and psychological factors. |
| Bennell et al. (2012)  PMID  23231928 | Randomized controlled trial protocol | N=168 | To investigate the clinical- and cost-effectiveness of adding telephone coaching to a physiotherapist-delivered physical activity intervention for people with knee OA. | Randomly allocated to physiotherapy only, or physiotherapy plus nurse-delivered telephone coaching. | Primary outcomes are pain and self-reported physical function. Secondary outcomes include physical activity levels, quality-of-life, and potential moderators and mediators of outcomes including self-efficacy, pain coping and depression. Relative cost-effectiveness will be determined from health service usage and outcome data. |
| Brosseau et al. (2012)  PMID  23234575 | Randomized controlled trial | N=222 | To improve knowledge translation in order to influence individuals to adopt effective self-management interventions, such as an adapted walking program. | Randomized to one of three knowledge translation groups: 1) Walking and Behavioural intervention, 2) Walking intervention, 3) Self-directed control. | The three groups were equivalent when determining the effectiveness of knowledge uptake and improvements in quality of life and other clinical outcomes. |
| Coleman et al. (2008)  PMID  18778467 | Case series | N=79 | To determine whether a specific self-management program for people with osteoarthritis of the knee, implemented by health professionals could achieve and maintain clinically meaningful improvements. | 6-week disease (OA) and site (knee) specific self-management education program; conducted in a community health care setting and was delivered by health professionals. | Improvements in indices of pain, mental health and physical functioning were demonstrated from baseline to 12 months. |
| Cronan et al. (1998)  PMID  9830877 | Randomized controlled trial | N=363 | To determine whether experimentally developed social support, education about appropriate use of the health care system, and their combination are effective in reducing health care costs for people with osteoarthritis. | Randomly assigned to 1 of 3 intervention groups or to a control group. | The 3 interventions had nearly equal effects on health status and health care costs. Health care costs in the combined experimental groups were lower than those in the control group by $1,279/ participant/ year in year 3. |
| Crotty et al. (2009)  PMID  19486959 | Randomized controlled trial | N=152 | To evaluate the efficacy of a self-management support program including a 6 week self-management course, individualized phone support and goal setting in osteoarthritis patients on a waiting list for arthroplasty surgery. | Participants were randomized to a self-management program or to usual care. | The self-management program improved health-directed behaviours, skill acquisition and stiffness, although the observed effects were of modest size. There was no significant effect on pain, function or quality of life in the short term. |
| Eitzen et al. (2015)  PMID  25886499 | Randomized controlled trial – secondary analysis | N=45 | To compare stance phase gait characteristics in hip osteoarthritis patients with mild to moderate symptoms participating in a randomized trial with two interventions. | Patient education only or patient education followed by a 12-week supervised exercise therapy program. | Adding a 12-week supervised exercise therapy program to patient education did not induce changes in our selected biomechanical variables during the stance phase of gait, even when adjusting for poor compliance. |
| Fernandes et al. (2010)  PMID  20633669 | Randomized controlled trial | N=109 | To compare the efficacy of patient education and supervised exercise with that of patient education alone for the management of pain in patients with hip osteoarthritis. | Patient education (PE). Patient education and supervised exercise (PE+SE). | No significant between group differences were found for pain over the 16-month follow-up. |
| Hartman et al. (2000)  PMID  11129742 | Randomized controlled trial | N=33 | To determine the effects of T'ai Chi training on arthritis self-efficacy, quality of life indicators, and lower extremity functional mobility in older adults with osteoarthritis. | T'ai Chi training included two 1-hour T'ai Chi classes per week for 12 weeks. | T'ai Chi participants experienced significant improvements in self-efficacy for arthritis symptoms, total arthritis self-efficacy, level of tension, and satisfaction with general health status. |
| Hay et al. (2006)  PMID  17056608 | Randomized controlled trial | N=325 | To evaluate the effectiveness of two primary care strategies for delivering evidence based care to people aged 55 or over with knee pain. | Enhanced pharmacy review (pharmacological management in accordance with an algorithm); community physiotherapy (advice about activity and pacing and an individualised exercise programme); control (advice leaflet reinforced by telephone call). | Short term improvements in health outcomes, reduced use of non-steroidal anti-inflammatory drugs, and high patient satisfaction. Physiotherapy seemed to produce a shift in consultation behaviour away from the traditional general practitioner led model of care. |
| Hughes et al. (2004)  PMID 15075418 | Randomized controlled trial | N=150 | To assess the impact of a low cost, multicomponent physical activity intervention for older adults with lower extremity osteoarthritis. | The training program consisted of range of motion, resistance training, aerobic walking, and education-group problem solving regarding self-efficacy for exercise and exercise adherence, versus wait list control group. | Individuals in the exercise program experienced an improvement in exercise efficacy, a 48.5% increase in exercise adherence, and a 13.3% increase in 6-min distance walk that were accompanied by significant decreases in lower extremity stiffness at 2 and 6 months. |
| Hurley et al. (2016)  PMID  26801470 | Randomized controlled trial protocol | TBD | To support an increase in self-management behaviour in patients with CLBP and OA in primary care physiotherapy. | Self-management of Osteoarthritis and Low back pain through Activity and Skills (SOLAS) intervention or usual individual physiotherapy. | The primary outcomes are the (1) acceptability and demand of the intervention to patients and physiotherapists, (2) feasibility and optimal study design/ procedures and sample size for a definitive trial. |
| Keefe et al. (1996)  PMID  8997917 | Randomized controlled trial | N=88 | To evaluate the effects of a spouse-assisted pain-coping skills training intervention on pain, psychological disability, physical disability, pain-coping, and pain behavior in patients with osteoarthritis of the knees. | Randomly assigned to 1 of 3 conditions: 1) spouse-assisted pain-coping skills training, 2) a conventional CST intervention with no spouse involvement, or 3) an arthritis education-spousal support control condition. | Patients in the spouse-assisted CST condition had significantly lower levels of pain, psychological disability, and pain behavior, and higher scores on measures of coping attempts, marital adjustment, and self-efficacy than patients in the control condition. |
| Kim et al. (2012)  PMID  21193289 | Nonequivalent control group, pre- and posttest quasi-experimental design | N=70 | To determine the effectiveness of an aquarobic program on weight control, lipid profiles, and levels of self-efficacy, pain, and depression with the intent to apply the program as a nursing intervention. | The aquarobic exercise program consisted of both patient education and aquarobic exercise. | The program was effective in enhancing self-efficacy, decreasing pain, and improving depression levels, body weight, and blood lipid levels in patients with osteoarthritis. |
| Lin, Davey & Cochrane (2004)  PMID  14763724 | Quasi-experimental design (exercise group and age-matched control group) | N=106 | To examine the effectiveness of a 12-month community-based water exercise program on measures of self-reported health and physical function in people aged over 60 years old with knee-hip osteoarthritis. | Participants in the exercise group were asked to attend two exercise sessions a week of 1 hour duration led by specially trained swimming instructors. Age-matched, non-exercising, 'control' subjects received monthly education material and quarterly telephone calls. | Older people with knee/hip osteoarthritis gained modest improvements in measures of physical function, pain, general mobility and flexibility after participating in 12 months of community-based water exercise. |
| Moe et al. (2010)  PMID  21040556 | Randomized controlled trial protocol | N= 400 | To compare the effects of a multidisciplinary outpatient clinic, including a brief group-based educational program, with a traditional individual outpatient clinic for patients with hip, knee, hand or generalized OA. A secondary purpose is to investigate the effects of a telephone follow-up call. | The experimental group is exposed to a multidisciplinary and multifaceted intervention, including a 3.5 hour group-based patient education program about OA in addition to individual consultations with members of a multidisciplinary team. The control intervention is based on regular care with an individual outpatient consultation with a rheumatologist (treatment as usual). | Primary outcomes are patient satisfaction measured at 4 months and cost-effectiveness measured at 12 months. Secondary outcomes are pain and global disease activity, generic and disease specific functioning and disability. |
| Østerås et al. (2014)  PMID  24629063 | Randomized controlled trial protocol | N=150 | To determine the effect of an exercise intervention on self-reported hand activity performance in people with hand OA. | The control group receives usual care, whereas the intervention group receives a 12-week exercise intervention. The intervention group attends four group sessions and is instructed to perform the exercise program three times a week at home. | The primary outcome measure is self-reported hand activity performance at 3 months post-randomization and a patient-generated measure of disability. Secondary outcome measures are self-reported OA symptoms, disease activity, measured hand function, and health-related quality of life. |
| Patel, Walsh &Gooberman-Hill (2014)  PMID 24840914 | Qualitative study (semi-structured interviews) | N=20 | To explore healthcare professionals' views on a group-based exercise intervention. | Group-based exercise intervention designed to facilitate the self-management of OA in the lower limbs and/or lower back. | Three themes were identified: 1) Patient understanding of osteoarthritis; 2) Multiple-joint approach and 3) Practical aspects of the intervention. Healthcare professionals saw the intervention as an acceptable and feasible approach to facilitate the self-management of OA. |
| Rosemann et al. (2007)  PMID  18050178 | Cluster-randomized controlled trial | N=1021 | To assess whether providing information on arthritis self-management through general practitioners (GPs) increases the quality of life in patients with osteoarthritis and whether additional case management provided by practice nurses shows better results. | GPs were randomized to intervention group I, group II, or a control group. GPs of both intervention groups participated in 2 peer group meetings. In intervention group II, additional case management was conducted via telephone by a practice nurse. | Intervention group I: radiographs decreased significantly, whereas prescriptions of acetaminophen increased significantly. Intervention group II: significant changes in the AIMS2-SF dimensions social, symptom, and lower body; radiographs and orthopedic referrals decreased whereas prescriptions of pain relievers increased significantly. |
| Skou et al.  PMID  23290290 | Pilot (cohort) study | N=36 | To examine the feasibility of the Good Life with osteoArthritis in Denmark (GLA:D) in persons with mild to moderate knee and/or hip OA-related pain. | The treatment consisted of two 1.5-hour sessions of patient education and six weeks of individualized supervised neuromuscular exercise according to the previously published NEuroMuscular Exercise programme. | There were significant improvements in pain, in time in the 20-meter walk test, in EQ-5D, in ASES and in the number of complete chair stands. Compliance was high in relation to both patient education and exercise. |
| Skou et al. (2014)  PMID 25123117 | Cohort study | N=79 | To identify predictors of effectiveness at one year from education and exercise in patients with knee or hip pain in clinical practice. | Good Life with Arthritis in Denmark is an implementation initiative consisting of education and 12 sessions of neuromuscular exercise delivered by trained physiotherapists. | Improvements in pain and EQ-5D at three months were maintained at one year. Change in self-efficacy from baseline to three months and 30-second chair stand test and self-efficacy at three months were predictors of one-year improvement in pain. Self-efficacy at three months was a predictor of one-year improvement in EQ-5D. |
| Stener-Victorin, Kruse-Smidje& Jung (2004)  PMID  15100594 | Randomized controlled trial | N=45 | To evaluate the therapeutic effect of electro-acupuncture and hydrotherapy, both in combination with patient education or with patient education alone, in the treatment of osteoarthritis in the hip. | Randomly allocated to electro-acupuncture, hydrotherapy, both in combination with patient education, or patient education alone. | EA and hydrotherapy, both in combination with patient education, reduced pain and ache and increased functional activity and quality of life. There were no changes in the education group alone. |
