# Supplementary material for: Education and Social Support as Key Factors in Osteoarthritis Management Programs: A Scoping Review
Source: Arthritis. 2018 May 8;2018:2496190. doi: 10.1155/2018/2496190 (PMC5964569; doi:10.1155/2018/2496190)
Supplement: Supplementary 2 — Supplementary Table 2: summary of included studies by community setting, education focus, and social support. [file 2496190.f2.docx]

**Supplementary Table 2.** Summary of included studies by community setting, education focus, and social support.

| Authors | Title | Year | Community-based | Education | Social Support |
| --- | --- | --- | --- | --- | --- |
| Alkatan et al. | Effects of swimming and cycling exercise intervention on vascular function in patients with osteoarthritis. | 2016 | Setting: community recreation/fitness arena | Focus: OA-specific  Topic: exercise training with active coaching and instruction | Positive social interaction From: peers (group-based)  Duration: 12 weeks |
| Bennell et al. | A physiotherapist-delivered integrated exercise and pain coping skills training intervention for individuals with knee osteoarthritis: a randomised controlled trial protocol. | 2012 | Setting: metropolitan private practices + home-based | Focus: OA-specific  Topic: pain coping skills training | Emotional/Informational From: physiotherapists  Duration: 10 weeks |
| Bennell et al. | Addition of telephone coaching to a physiotherapist-delivered physical activity program in people with knee osteoarthritis: A randomized controlled trial protocol. | 2012 | Setting: metropolitan and regional private practices + home-based | Focus: OA-specific  Topic: exercise and self-management | Emotional/Informational From: physiotherapists, registered nurses  Duration: 24 weeks |
| Brosseau et al. | The implementation of a community-based aerobic walking program for mild to moderate knee osteoarthritis: a knowledge translation randomized controlled trial: part II: clinical outcomes. | 2012 | Setting: walking club sites | Focus: arthritis  Topic: adapted *Program for Arthritis Control through Education and Exercise* (PACEex) program | Positive social interaction, Emotional/Informational From: peers (group-based), trained instructor  Duration: 52 weeks |
| Coleman et al. | Short and medium-term effects of an education self-management program for individuals with osteoarthritis of the knee, designed and delivered by health professionals: a quality assurance study. | 2008 | Setting: community health care setting | Focus: knee OA-specific  Topic: self-management | Positive social interaction From: peers (group-based)  Duration: 6 weeks |
| Cronan et al. | The effects of social support and education on health care costs after three years. | 1998 | Setting: health maintenance organizations | Focus: OA-specific  Topic: self-management | Positive social interaction From: peers (group-based)  Duration: 50 weeks |
| Crotty et al. | Self-management and peer support among people with arthritis on a hospital joint replacement waiting list: a randomised controlled trial. | 2009 | Setting: public hospitals | Focus: arthritis  Topic: Flinders University Chronic Disease Self-Management Model | Positive social interaction,  Emotional/Informational From: peer support volunteers, specialist nurse  Duration: 6 weeks |
| Eitzen et al. | No effects of a 12-week supervised exercise therapy program on gait in patients with mild to moderate osteoarthritis: a secondary analysis of a randomized trial. | 2015 | Setting: rehabilitation center | Focus: hip OA-specific  Topic: self-management and exercise therapy program | Positive social interaction,  Emotional/Informational From: peers (group-based), physical therapist  Duration: 12 weeks |
| Fernandes et al. | Efficacy of patient education and supervised exercise vs patient education alone in patients with hip osteoarthritis: a single blind randomized clinical trial. | 2010 | Setting: rehabilitation center | Focus: hip OA-specific  Topic: self-management and exercise therapy program | Positive social interaction,  Emotional/Informational From: peers (group-based), physical therapist  Duration: 12 weeks |
| Hartman et al. | Effects of T'ai Chi training on function and quality of life indicators in older adults with osteoarthritis. | 2000 | Setting: college | Focus: lower extremity OA-specific  Topic: T'ai Chi training | Positive social interaction From: peers (group-based)  Duration: 12 weeks |
| Hay et al. | Effectiveness of community physiotherapy and enhanced pharmacy review for knee pain in people aged over 55 presenting to primary care: pragmatic randomised trial. | 2006 | Setting: general practices | Focus: knee OA-specific  Topic: Arthritis Research Campaign information leaflet and exercise training | Emotional/Informational From: a pharmacist and physiotherapists  Duration: 10 weeks |
| Hughes et al. | Impact of the fit and strong intervention on older adults with osteoarthritis. | 2004 | Setting: senior centers and senior housing residences | Focus: lower extremity OA-specific  Topic: exercise and self-efficacy training | Positive social interaction,  Emotional/Informational From: peers (group-based), physical therapists  Duration: 8 weeks (+ 104 weeks follow-up) |
| Hurley et al. | Theory-driven group-based complex intervention to support self-management of osteoarthritis and low back pain in primary care physiotherapy: protocol for a cluster randomised controlled feasibility trial (SOLAS). | 2016 | Setting: primary community and continuing care clinics | Focus: chronic conditions  Topic: Self-management of OA and LBP through Activity and Skills (SOLAS) | Positive social interaction,  Emotional/Informational From: peers (group-based), physiotherapists  Duration: 6 weeks |
| Keefe et al. | Spouse‐assisted coping skills training in the management of osteoarthritic knee pain. | 1996 | Setting: unclear [attendance at weekly sessions] | Focus: arthritis  Topic: pain coping skills training | Positive social interaction,  Emotional/Informational From: spouses, peers (group-based), psychologists, nurses  Duration: 10 weeks |
| Kim et al. | The effectiveness of an aquarobic exercise program for patients with osteoarthritis. | 2012 | Setting: unclear  [local health care center with swimming facility] | Focus: OA-specific  Topic: exercise training | Positive social interaction,  Emotional/Informational From: peers (group-based), aquarobic instructor  Duration: 12 weeks |
| Lin, Davey & Cochrane | Community rehabilitation for older adults with osteoarthritis of the lower limb: a controlled clinical trial. | 2004 | Setting: public community swimming pool | Focus: lower extremity OA-specific  Topic: water exercise program | Positive social interaction,  Emotional/Informational From: peers (group-based), swimming instructors  Duration: 46 weeks |
| Moe et al. | Multidisciplinary and multifaceted outpatient management of patients with osteoarthritis: protocol for a randomised, controlled trial. | 2010 | Setting: outpatient hospital rheumatology clinic | Focus: OA-specific  Topic: self-management | Emotional/Informational From: researchers  Duration: follow-up at 16 weeks (phone call) |
| Østerås et al. | Exercise programme with telephone follow-up for people with hand osteoarthritis–protocol for a randomised controlled trial. | 2014 | Setting: primary health care centre and hospital | Focus: hand OA-specific  Topic: exercise training | Positive social interaction,  Emotional/Informational From: peers (group-based), physiotherapists, occupational therapists  Duration: 12 weeks |
| Patel, Walsh & Gooberman-Hill | Managing osteoarthritis in primary care: exploring healthcare professionals’ views on a multiple‐joint intervention designed to facilitate self‐management. | 2014 | Setting: primary care | Focus: multiple joints, OA-specific  Topic: generic self-management and exercise training (FASA programme) | Positive social interaction,  Emotional/Informational From: peers (group-based), physiotherapist  Duration: 6 weeks |
| Rosemann et al. | Case management of arthritis patients in primary care: A cluster‐randomized controlled trial. | 2007 | Setting: primary care | Focus: arthritis  Topic: self-management | Emotional/Informational From: practice nurses  Duration: unclear (phone call every 4 weeks) |
| Skou et al. | Group education and exercise is feasible in knee and hip osteoarthritis. | 2012 | Setting: unclear  [physiotherapy clinic] | Focus: lower extremity OA-specific  Topic: Good Life with osteoArthritis in Denmark (GLA:D) self-management and neuromuscular exercise training | Positive social interaction,  Emotional/Informational From: peers (group-based), physiotherapists, dietician, peer leader (patient)  Duration: 6 weeks |
| Skou et al. | Predictors of long-term effect from education and exercise in patients with knee and hip pain. | 2014 | Setting: physiotherapy clinic | Focus: lower extremity OA-specific  Topic: Good Life with osteoArthritis in Denmark (GLA:D) self-management and neuromuscular exercise training | Positive social interaction,  Emotional/Informational From: peer leader (previous GLA:D participant), physiotherapists  Duration: 8 weeks |
| Stener-Victorin, Kruse-Smidje & Jung | Comparison between electro-acupuncture and hydrotherapy, both in combination with patient education and patient education alone, on the symptomatic treatment of osteoarthritis of the hip. | 2004 | Setting: physiotherapy clinic | Focus: hip OA-specific  Topic: self-management | Positive social interaction,  Emotional/Informational From: peers (group-based), physiotherapists  Duration: 5 weeks |
